# C-Reactive Protein (CRP): A poor prognostic biomarker in COVID-19

**DOI:** 10.3389/fimmu.2022.1040024

**Published:** 2022-11-14

**Authors:** Mohamed Zakaria Bouayed, Ilyass Laaribi, Charaf Eddine Mohammed Chatar, Iliass Benaini, Mohammed Amine Bouazzaoui, Younes Oujidi, Samia Berrichi, Ghizlane El Aidouni, Houssam Bkiyar, Naima Abda, Brahim Housni

**Affiliations:** ^1^ Anesthesia, Intensive Care and Resuscitation Department, MOHAMMED VI University Hospital, Oujda, Morocco; ^2^ Laboratory of Epidemiology, Clinical Research, and Public Health, Faculty of Medicine and Pharmacy, Mohammed First University, Oujda, Morocco; ^3^ Simulation Center, Faculty of Medicine and Pharmacy, Mohammed First University, Oujda, Morocco

**Keywords:** COVID-19, C Reactive Protein (CRP), SARS-CoV-2, biomarkers, prognosis, mortality

## Abstract

**Introduction:**

The COVID-19 pandemic continues to be rampant with considerable morbidity and mortality worldwide since its emergence in December 2019. Several studies have focused on identifying different predictive factors of poor prognosis, including biological markers, such as C Reactive Protein among others. The objective of our work was to determine whether the CRP levels on admission to the intensive care unit are predictive of an unfavorable evolution of patients with COVID-19 through the experience of the Anesthesia and Intensive Care Unit of the University Hospital of Oujda and to compare our results with those reported in the literature.

**Methods:**

We conducted a retrospective, monocentric, descriptive and analytical study in the Department of Anesthesia and Intensive Care of the Mohammed VI University Hospital of Oujda, Morocco, between March 2020 and October 2021, including all critically ill patients admitted to the department during this period and meeting the inclusion criteria. The baseline admission CRP value was arbitrarily set at 100mg/d, thus conditioning the division of our patients into two groups (group 1: CRP < 100mg/L, group 2: CRP ≥ 100mg/L).

**Results:**

Among our 1035 included patients, 291 patients with had a CRP<100mlg/L (group 1) and 744 presented a CRP level equal or superior to 100mg/L (group 2). Lung parenchymal involvement was more severe or even critical (CT involvement > 75%) in group 2 (60.8%) compared to group 1 (39.2%). In group 2, 79.8% of patients were mechanically ventilated, compared to 20.2% of patients in group 1. Finally, the mortality rate in patients with a CRP ≥ 100mg/l was 77.4%, compared with 22.6% for patients with a CRP < 100mg/l. These findings are all statistically highly significant (p<0.001)

**Conclusion:**

Given the high contagiousness of the virus and the emergence of several variants, the management of the COVID-19 pandemic has focused more on prevention through vaccination against the virus, but also on an early identification of patients likely to evolve unfavorably for a personalized management.

## Introduction

Since December 2019, the COVID-19 epidemic, now known to all, continues to be rampant, with the number of infected cases increasing exponentially (1).

Complications of COVID-19 mainly involve the respiratory, renal and cardiovascular systems. In severe cases, secondary infections lead to pneumonia and acute respiratory distress syndrome, which may precede the patient’s death. Multi-organ failure in individuals with COVID-19 could be a consequence of decompensation of their comorbidities and/or deregulated immune response. A patient’s pre-existing conditions may affect the prognosis of the disease, requiring immediate attention to accurately detect and evaluate individuals infected with SARS-CoV-2 (2).

Several studies have noted an abnormal imbalance in the analytical data of different biological markers. These biomarkers include C-reactive protein (CRP), leukocytes, lymphocytes, platelets, D-dimers, Interleukin 6 IL-6 among others.

The objective of this study is to determine whether CRP levels on admission to the Intensive Care Unit are predictive of an unfavorable evolution of patients with COVID-19 through the experience of the Anesthesia, Intensive Care, and Resuscitation Department of the Mohammed VI University Hospital of Oujda and to compare our results with those reported in the literature.

## Methods

### Type of study

This is a monocentric, descriptive and analytical retrospective cohort study carried out in the Department of Anesthesia and Intensive Care of the Mohammed VI University Hospital of Oujda, in collaboration with the Laboratory of Epidemiology, Clinical Research, and Public Health (LERCSP) of the Faculty of Medicine and Pharmacy of Oujda, conducted over a period of 19 months, between March 2020 and October 2021.

Inclusion criteria:

We included:

- Confirmed cases of COVID-19 defined by a positive reverse transcriptase polymerase chain reaction (RT-PCR) test of a nasopharyngeal swab sample.
- Patients at least 18 years of age
- Patients with a plasma C-reactive protein (CRP) bioassay on admission

Exclusion criteria:

We excluded:

- Patients with elevated CRP levels on admission explained by a pathology outside of COVID-19 (proven infection, neoplasia,…).
- Patients who were hospitalized for < 48 hours.
- Patients with missing or absent biological data.

Data collection and statistical analysis:

First, a descriptive analysis of the socio-demographic characteristics was performed to describe the study sample at inclusion. The results are expressed as numbers and percentages for the categorical variables and mean and standard deviation for the quantitative variables. A univariate analysis of the different variables was performed. The Chi-square test was used for categorical variables and the Student’s t-test for quantitative variables.

To study the relevance of initial CRP in predicting mortality, patients were divided into two groups according to a baseline admission CRP value set at 100mg/L, chosen based on the optimum cut-off value identified by drawing AUC curves. And the Cox proportional hazards model with “delayed entry” was used for analysis of right-censored cohort data and to account for potential confounders

All variables with an association with a significance level (p-value) <0.20 in the univariate analysis were included in the initial model. Then we proceeded with a manual top-down stepwise analysis with a threshold at 5%, eliminating each time the variable with the highest p-value. We retained in the final model only those variables with a p-value <0.05 for which an estimate of the relative risks and 95% confidence intervals was calculated; diabetes was forced in the final model given its relevance.

Statistical entry and analysis were performed using IBM SPSS Statistics software (Version 21.0).

### Ethical Considerations

This study is compliant with the World Medical Association’s Code of Ethics (Declaration of Helsinki) and was granted approval by the Ethics Committee for Biomedical Research of Oujda (N° 017/20)

Written informed consent was obtained from each participant.

This study is registered in the Research Registry under the number: 8281

### Results

#### Descriptive study

During the study period, of the 1112 patients hospitalized in our department for the management of a COVID-19 infection, 1035 patients met our inclusion criteria.

Demographics and baseline characteristics:

1035 cases were identified over 20 months, with an annual frequency of 647cases/year.

The mean age of our patients was 63.01 ± 17.03, ranging from 18 to 101 years.

The distribution of patients was as follows:

- 25 patients had an age between 18-39 years representing 2.5%.
- 272 patients were between 40-59 years of age representing 26.31%.
- 620 patients were between 60-79 years of age representing 59.86%.
- 118 patients were >80 years old representing 11.33%.

Men represented 62.8% of the cases studied (n=650), while women constituted 37.2% (n=385), with a M/F sex ratio of 1.68.

The frequency of comorbidities identified was variable, represented in **Table 1**.

**Table 1:** Summary of the demographic and baseline characteristics, laboratory/radiological findings, and Treatments/outcome of the patients.

Demographic and baseline characteristics.			
Mean age (+/- SD)			63.01 (+/-17.03)
Sex (%)		Male	650 (62,8%)
		Female	385 (37,2%)
Length of stay, days (+/-SD)			7,83 (+/-7,07)
Comorbidities, n (%)		Obesity (BMI > 30kg/m²)	167 (16,1%)
		Hypertension	337 (32,6%)
		Diabetes	343 (33,1%)
		Cardiopathy	141 (13,6%)
		Chronic renal failure	58 (5,6%)
		Pre-existing respiratory disease (asthma, COPD, silicosis…)	32 (3,1%)
		Cancer	11 (1,06%)
Symptoms, n (%)		Fever	890 (85,9%)
		Chills	436 (42,12%)
		Dyspnea	882 (85,2%)
		Cough	790 (76,55%)
		Anosmia	121 (11,69%)
		Headache	558 (53,91%)
		Asthenia	748 (72,27%)
		Digestive signs	370 35,74%)
Clinical assessment at admission		Consciousness, n (%): - GCS 15/15 - GCS between 9 and 14 - GCS ≤ 8/15	865 (83,6%) 113 (10,9%) 57 (5,5%)
		Blood pressure, n (%): - Normal - Low - Shock	587 (56,7%) 236 (22,8%) 212 (19,5%)
		Respiratory parameters, mean (+/-SD) - SpO_2_ - PaO_2_	88,09 (+/- 6,03) 66,16 (+/- 29,4)
**Laboratory and radiological findings.**			
Blood cell counts			
High WBC, n (%)		456 (44,05%)	
Leucopenia, n (%)		51 (4,92%)	
Thrombocytosis, n (%)		302 (29,17%)	
Thrombocytopenia, n (%)		182 (17,58%)	
Lymphocytopenia		897 (86,7%)	
Inflammation biomarkers			
CRP, n (%)	< 100mg/L	291 (28,1%)	
	≥ 100mg/L	744 (71,9%)	
Ferritin, mean (range) (µg/L)		1742,2 (10 – 4000)	
High ferritin level, n (%)		940 (90,8%)	
Procalcitonin, mean (range) (ng/L)		207,17 (0,12 – 692)	
High procalcitonin level, n (%)		345 (33,3%)	
Interleukin 6, mean (range) (pg/mL)		194,82 (0,35 – 6000)	
High IL-6 level, n (%)		901 (87,05%)	
Hemostasis markers			
Prothrombin time, mean (+/-SD) (%)		76,15±18,29 (17 – 98)	
D-dimers, mean (range) (mg/L)		7,23 (0,31 – 45,20)	
High d-dimers level, n (%)		149 (14,39%)	
Fibrinogen, mean (+/-SD) (g/L)		5.25 ±2,79 (0,41 – 9,10)	
High fibrinogen level, n (%)		952 (91,98%)	
Other markers			
High lactate dehydrogenase level, n (%)		931 (89,95%)	
**Radiological findings**			
Degree of pulmonary involvement in CT, n (%)		Critical (≥75%)	362 (35,8%)
		Severe (50 – 75%)	340 (33,6%)
		Mild (25 – 50%)	214 (21,1%)
		Low (< 25%)	96 (9,5%)
Pulmonary embolism, n (%)		58 (5,73%)	
**Treatments and Outcome.**			
Oxygen support, n (%)			
Nasal oxygen therapy		588 (56,8%)	
High concentration oxygen therapy		717 (85,71%)	
High flow nasal oxygen therapy		497 (48%)	
Continuous positive airway pressure (CPAP) therapy		75 (7,24%)	
Noninvasive ventilation (NIV)		288 (27,8%)	
Mechanical ventilation (MV)		259 (25%)	
Veno-venous Extracorporeal membrane oxygenation (VV-ECMO)		28 (2,7%)	
Corticosteroids therapy, n (%)			
Methylprednisolone		576 (55,64%)	
Dexamethasone		131 (12,65%)	
Hydrocortisone		36 (3,47%)	
Anticoagulation, n (%)			
Enoxaparine		971 (95,6%)	
Tinzaparine		44 (4,4%)	
Platelet anti-aggregation inhibitors, n (%)			
Acetylsalicylic acid		816 (78,84%)	
Anti-interleukin-6 therapy			
Tocilizumab		191 (18,45%)	
Antibiotics, n (%)			
Amoxicillin + clavulanic acid		208 (32,2%)	
Ceftriaxone		289 (44,7%)	
Ceftriaxone + ciprofloxacin		92 (14,4%)	
Piperacillin-Tazobactam + voriconazole + amikacin		59 (8,7%)	
Adjuvant therapies, n (%)			
Vitamin C		1035 (100%)	
Vitamin D		1035 (100%)	
Zinc		1035 (100%)	
Proton pump inhibitors		1035 (100%)	
Outcome, n (%)			
Survival		685 (66,2%)	
Death		350 (33,8%)	

The average number of hospitalization days was 7.83 ± 7.07 with extremes ranging from 2 to 49 days.

Patients complained of a number of symptoms. An initial clinical assessment of the neurological, hemodynamic and respiratory state was performed at admission (**Table 1**).

#### Laboratory and radiological findings

Upon admission, all 1035 cases underwent laboratory testing, the results of which are presented in **Table 1**. The most observed biological abnormalities are: lymphocytopenia (86,7%), hyperferritinemia (90,8%), high IL-6 levels (87,05%), hyperfibrinogemia (91,98%), high LDH levels (89,95%).

As for radiological findings, the pulmonary involvement was graded according to the CO-RADS classification system as demonstrated in **Table 1**. Contrast chest CT, when performed, revealed a pulmonary embolism in 5,73% of cases.

#### Treatments and Outcome

Throughout their hospital stay, patients underwent various treatment shown in **Table 1**. Oxygen supplementation varied from a patient to another according on each patient’s needs, sometimes requiring a more proficient oxygen or ventilation method depending on patients’ response and evolution. As for the medication, the protocol relied mainly on anticoagulation (100%), platelets anti-aggregation therapy (78,84%), corticosteroids mainly methylprednisolone (55,64%), adjuvant therapies (Vitamins C and D, Zinc), as well as antibiotics.

A favorable outcome was noted in 66,2% of cases as demonstrated in **Table 1**.

### Analytical study

**Figure 1:**
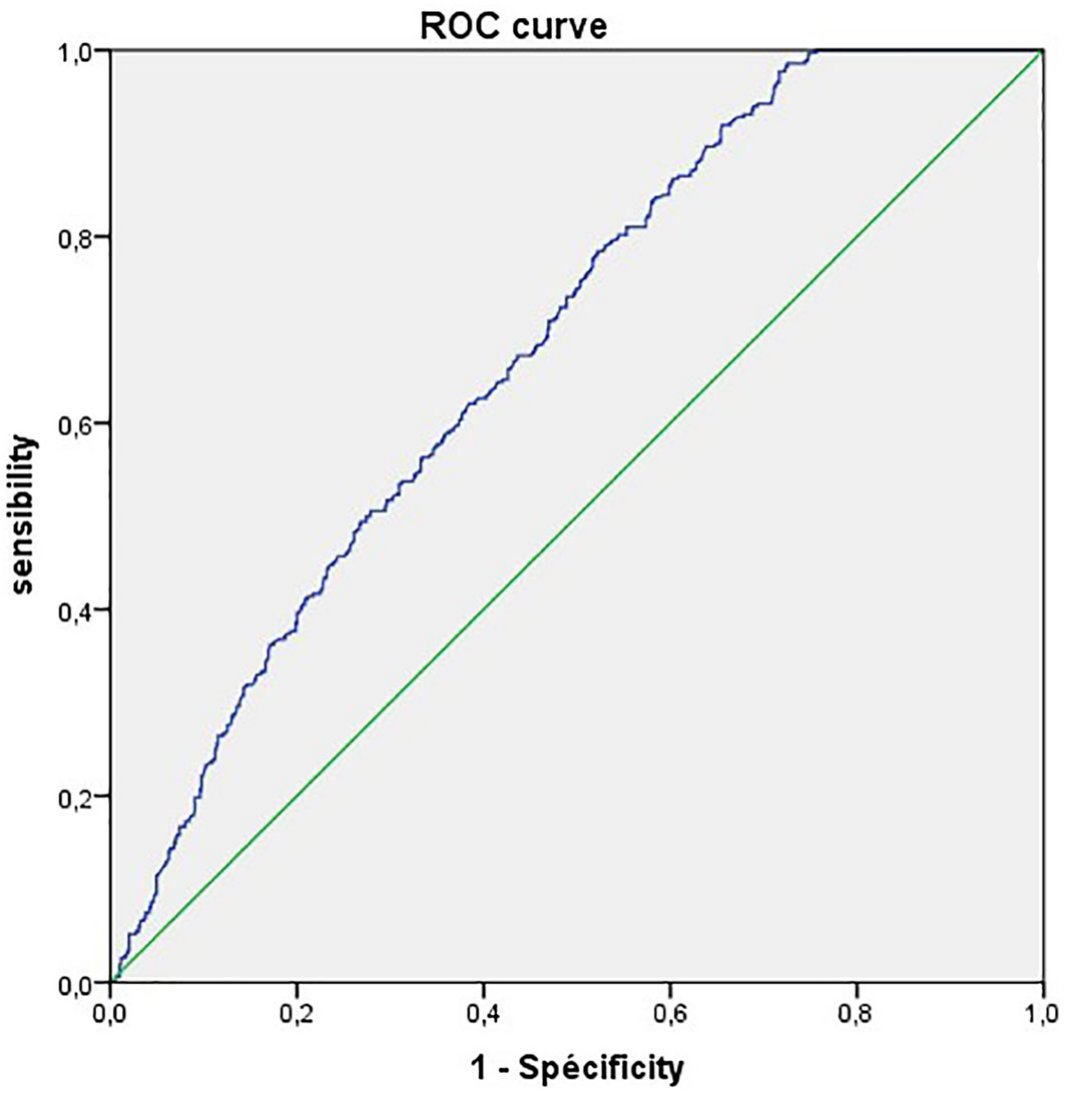
Receiver operating characteristic (ROC) curves of CRP for predicting the disease severity in COVID-19.

To provide statistical evidence for the considered CRP threshold, we performed a ROC curves analyses (**Figure 1**).

The ROC curve analysis finds an AUC of 68.2%. with a CRP cut-off value of 100mg/L we obtained the highest sensitivity of 88.5%.

Of the 1035 cases, 291 patients (28,1%) were allocated to the nonsevere group (group 1) given a CRP level below 100mg/L, while 744 patients (71,9%) were assigned to the severe group (group 2) given their CRP levels equal or higher than 100mg/L.

We noticed that the mean age of patients with CRP < 100 mg/l (group1) is 60.39 years while the mean age in patients with CRP ≥ 100mg/l (group2) is 64.33 years, this correlation was statistically proven (p= <0.001). as for the obesity rate, it was calculated at 25.4% in patients with CRP <100 mg/l (group 1), and 74.6% in patients with CRP≥100mg/l (group 2), thus not statistically significant (p= 0.080). We also found that hypertensive patients represented 27.6% in group 1 and 72.4% in group 2, which is also not statistically significant (p= 0.09). Meanwhile, diabetic patients represented 27% in group 1 and 73% in group 2, this correlation is statistically significant (p= 0.046). As for heart disease (all types), it was found in 34.4% of group 1 and 65.6% of group 2, which is not statistically significant (p= 0.329).

We found that the mean of pulsed Oxygen saturation in group 1 was 90.45%, vs 81.80% in group 2, a correlation that is statistically significant with a p value < 0.001.

The mean WBC count in patients with CRP <100 mg/l is 12,676×10 (3)/μL, whereas for the second group (CRP≥100mg/l), the mean WBC count is 12,442×10 (3)/μL, an association that was not statistically significant, with a p-value=0.08. we also noticed that the mean of LDH level in group 1is 591.57 IU/L vs 736.64 IU/L for group 2, which is statistically significant with a p value < 0.001. As for ferritin levels, the mean in group 1 was 1392.69 µg/L and 1940.86µg/L in group 2, an association that is statistically proven with a p value at 0.03.

The degree of pulmonary involvement was more severe, even critical (CT involvement > 75%) in group 2 compared with group 1 and statistically significant with a P value < 0.001.

In group 2, 79.8% of patients were mechanically ventilated, compared to 20.2% of patients in group 1, this correlation is also statistically significant with a P value < 0.001.

We found that the mortality rate in patients with CRP ≥ 100mg/l is 77.4%, compared with 22.6% for patients with CRP < 100mg/l with a statistical significance (P value < 0.001).

The univariate analysis is summarized in **Table 2**.

**Table 2:** Univariate and multivariate analyses of the logistic regression model.

Variable	Univariate analysis			Multivariate analysis	
	Group 1 (n = 291) (CRP < 100mg/L)	Group 2 (n= 744) (CRP ≥100mg/L)	p value	Odds ratio (95% CI)	p value
Age, mean	60,39	64,33	< 0,001	1,017 (1.009 - 1.026)	< 0,001
Obesity, n (%)	263 (25,4%)	772 (74,6%)	0.080		
Hypertension, n (%)	286 (27,6%)	749 (72,4%)	0.09		
Diabetes	279 (27%)	756 (73%)	0.046	1,184 (0.941 – 1.490)	0,149
Cardiopathy	356 (34,4%)	679 (65,6%)	0.329		
SpO_2,_ mean (%)	90,45%	81,80%	< 0,001	0,988 (0.978 - 0.998)	0,016
WBC, mean (×10^3^/μL)	12,676	12,442	0.08	1,000 (1.000 – 1.000)	< 0,001
LDH level, mean (IU/L)	591,57	736,64	< 0,001		
Ferritin level, mean (µg/L)	1392,69	1940,86	0.03	1,000 (1.000 – 1.000)	0,005
Critical lung involvement on CT, n (%)	142 (39,2%)	220 (60,8%)	< 0,001		
Mechanical ventilation, n (%)	66 (25,4%)	193 (74,6%)	< 0,001	2,288 (1.807 – 2.897)	< 0,001
Deaths, n (%)	79 (22,6%)	271 (77,4%)	< 0.00,1		

We performed a multivariate study to determine the different clinical and biological parameters significantly associated with mortality: Advanced age is a risk factor for mortality with a p=0.001, OR=1.017 [1.009 - 1.026]. Respiratory unstable patients with low pulse oxygen saturation are at higher risk of mortality with a p= 0.016, OR= 0.988 [0.978 - 0.998]. Also, high mortality is significantly associated with disturbed biological inflammatory values, notably C-reactive protein, ferritinemia and WBC with a P=0.016, OR=1.541 [1.082 - 2.194]; P=0.05, OR=1[1.000 - 1.000] and P=0.001, OR= 1[1.000 - 1.000] respectively. As for mechanical ventilation, it is associated with a higher mortality rate with a P=0.001, OR=2.288 [1.807 - 2.897]. Diabetes was added to these factors, all predisposing to a high mortality rate, but as a forced factor with a P=0.149, OR=1.148 [0.941 - 1.490], since it is pertinently reported by the literature data.

The multivariate analysis is summarized in **Table 2**.

## Discussion

The rationale behind any study aiming to identify prognostic factors in any given disease is to ultimately identify patients with the highest risks of unfavorable outcome and act accordingly.

The COVID-19 pandemic has proven to be a challenging experience of the health professionals worldwide as its definition progressed from a viral pneumonia to a systemic disease with multi-organ failure accredited to the disproportionate immune response also known as the cytokine storm (3).

This redefinition implicated a new approach in not only identifying factors incriminated in the unfavorable progression of the disease most particularly biomarkers indicative of the hyperinflammatory state (4), but most importantly in choosing the right therapy especially with the increasing role of immunosuppressive therapies and transfusion-based blood purification therapies such as convalescent plasma or plasma exchange (5).

C-reactive protein (CRP) is an acute-phase pentameric protein, its synthesis takes place in the liver, mainly induced by interleukin-6, in response to inflammation, having both pro- and anti-inflammatory properties, it is a key player in the process of recognition and clearance of foreign pathogens (6).

CRP concertation has a history of being associated with the severity of viral infections such as H1N1 influenza pneumonia (7), and has been pointed out early-on as a severity indicator of COVID-19 as shown in a systemic review by Ikeagwulonu et al (8) which included 61 studies with a total of 13891 COVID-19 patients, demonstrating that severe cases had constantly higher levels of CRP compared to mild cases, and that the increase in C-reactive protein was statistically significant in 78.7% of the cases included.

In a cohort conducted by Smilowitz et al (9), including 2782 COVID-19 patients, CRP levels above 108mg/L we associated with disease severity (47,6% vs 25,9%) and a higher mortality (32,2% vs 17,8%). Similarly, in a retrospective study conducted by Sadeghi et al (10). including 429 patients, it has been shown that not only the severe cases had significantly higher CRP levels than nonsevere patients, but also that patients with CRP >64.75 mg/L were more likely to have severe complications.

Similarly to our study, Luo et al (11) conducted a single center retrospective cohort enrolling 298 patients over a one-year period and aiming to determine the prognostic value of admission CRP. They concluded that not only CRP was a discriminator of severe/critical illness on admission but also independent predictor of unfavorable outcome, with a cut-off value of 41,4 exhibiting the highest sensitivity of 95,4%.

In a wider literature review conducted by Tjendra et al. (12) of multiple biomarkers incriminated in predicting COVID-19 severity, elevated inflammatory markers including elevated CRP was associated with a higher risk of disease progression to critical illness, a severe disease course, a higher risk of developing sepsis with rapid progression, and ultimately a higher risk of intubation and in-hospital mortality.

In the present study, multiple clinical, radiological, and biological characteristics of COVID-19 patients hospitalized within the specified timeline in our center were compared between severe and nonsevere patients in order to identify the factors associated with unfavorable disease progression and severity.

Our findings indicated that patients with a CRP level ≥ 100 mg/L were more likely to develop a severe form of the disease, and are more likely to die, thus conforming that CRP is a reliable predictor of COVID-19’s severity.

Although being a nonspecific inflammatory biomarker, C Reactive Protein as a prognostic factor is particularly interesting given its wide availability and frequent dosage compared to other biomarkers.

## Conclusion

Based on the premise that hyperinflammation is the main cause of critical forms of COVID-19 (13, 14), it is paramount to identify independent factors reflecting the extent of said inflammatory state and more precisely those that can be used to identify the most severe cases in order to manage them more promptly. Also, given that our patients underwent a similar protocol treatment with some adjustments to every case, we can safely presume that C Reactive Protein can be used to predict the cases that are more susceptible to progress unfavorably

In the prospect of anticipating COVID-19’s unfavorable progression, our study aimed to determine whether C- Reactive Protein can be used as a reliable prognostic indicator to that effect. Our findings show that a CRP value of ≥100mg can be correlated to the disease’s severity as well as a high risk of mortality.

## Data availability statement

The original contributions presented in the study are included in the article/supplementary material. Further inquiries can be directed to the corresponding author.

## Ethics statement

This study is compliant with the World Medical Association's Code of Ethics (Declaration of Helsinki) and was reviewed and approved by the Ethics Committee for Biomedical Research of Oujda ECBRO (N° 017/20). The participants provided their written informed consent to participate in this study. This study is registered in the Research Registry under the number: 8281.

## Author contributions

MZB and IL: Co-first authors, study conception, data collection; data analysis; writing & editing. MCEC: Data collection and analysis; writing & editing. IB: Writing and editing. MAB: data analysis. YO: Contributor. SB: Contributor. GE: Contributor. HB: Supervision and review, data validation. NA: Supervision and review, data validation. BH: Supervision and review, data validation. All authors contributed to the article and approved the submitted version.

## Conflict of interest

The authors declare that the research was conducted in the absence of any commercial or financial relationships that could be construed as a potential conflict of interest.

## Publisher’s note

All claims expressed in this article are solely those of the authors and do not necessarily represent those of their affiliated organizations, or those of the publisher, the editors and the reviewers. Any product that may be evaluated in this article, or claim that may be made by its manufacturer, is not guaranteed or endorsed by the publisher.
